# A Stochastic Nash Equilibrium Problem for Medical Supply Competition

**DOI:** 10.1007/s10957-022-02025-y

**Published:** 2022-04-10

**Authors:** Georgia Fargetta, Antonino Maugeri, Laura Scrimali

**Affiliations:** University of Catania, Catania, Italy

**Keywords:** Stochastic programming problem, Variational inequality, Duality, Medical Supplies, 49J40, 90C46, 90B15

## Abstract

In this paper, we study the competition of healthcare institutions for medical supplies in emergencies caused by natural disasters. In particular, we develop a two-stage procurement planning model in a random environment. We consider a pre-event policy, in which each healthcare institution seeks to minimize the purchasing cost of medical items and the transportation time from the first stage, and a recourse decision process to optimize the expected overall costs and the penalty for the prior plan, in response to each disaster scenario. Thus, each institution deals with a two-stage stochastic programming model that takes into account the unmet demand at the first stage, and the consequent penalty. Then, the institutions simultaneously solve their own stochastic optimization problems and reach a stable state governed by the stochastic Nash equilibrium concept. Moreover, we formulate the problem as a variational inequality; both the discrete and the general probability distribution cases are described. We also present an alternative formulation using infinite-dimensional duality tools. Finally, we discuss some numerical illustrations applying the progressive hedging algorithm.

## Introduction

Emergencies resulting from man-made or natural events strongly affect our social and economic life. Depending on the type of emergency different hazards may occur in the emergency locations. Thus, emergency management has raised increasing interest. In particular, businesses require special measures to protect their activities from any potential dangerous effect of an emergency. Therefore, it is important to establish a plan before the occurrence of these events to be prepared in case an emergency happens. A business continuity plan defines how a company will continue operating, even in the case of a natural disaster, IT failure or a cyber attack. The end goal is to preserve profitability and market position [12].

In this paper, we focus on a plan for the storage and distribution of medical supplies among healthcare institutions in emergencies caused by natural disasters. In particular, we model the competition among hospitals as a Nash equilibrium problem and introduce a stochastic programming model to design and evaluate the behavior of each demand location. Inspired by [26, 35], we provide a two-stage stochastic programming model based on disaster scenarios that introduces the unmet demand at the first stage and the consequent penalty at the second stage, see also [13]. Thus, we consider a pre-event policy, in which each healthcare institution minimize both the purchasing cost of medical items and the transportation time from the first stage. Then, we present a post-event policy through a recourse decision process to optimize the expected overall costs, and the penalty for the preassigned plan, in response to each possible disaster scenario of the second stage. Institutions simultaneously solve their own two-stage stochastic optimization problems and reach a stable state governed by the stochastic Nash equilibrium concept, that is formulated as a large-scale variational inequality. In addition, in the case of a general probability distribution, we define the stochastic Nash equilibrium as a random variational inequality in a Hilbert space setting. Then, we give the first order optimality conditions for the second-stage problem in terms of Lagrange multipliers, using a separation assumption, called *Assumption S*, as a constraint qualification [4–6, 9]. This condition results to be a necessary and sufficient condition for strong duality to hold. In infinite-dimensional spaces, the classical theorems, which prove strong duality and existence of multipliers, require that the interior of the ordering cone be nonempty [21]. However, in most infinite-dimensional cases, where the functional space is $L^2$ or a Sobolev space, the ordering cone has the empty interior. Therefore, we aim at proving that the second-stage problem verifies the *Assumption S*. As a result, we ensure the existence of Lagrange multipliers and give an alternative formulation of the two-stage problem. Moreover, we show that the dual variables regulate the medical item procurement. In fact, they represent the control variables on the first-stage demand, on the second-stage demand, and on the unfulfilled demand.

The importance of an efficient approach to emergency management and medical supply planning has been investigated in several papers. As an example, in [26], the authors presented a stochastic programming model, in which they selected the storage locations of medical supplies and use inventory levels for medical items. In the model, they captured the information updating during disaster scenarios. In [28], Nagurney et al. developed a stochastic generalized Nash equilibrium model consisting of multiple purchase locations for the disaster relief items, multiple humanitarian organizations, multiple freight service provision options and multiple hubs for storage to multiple points of demand. In [29], the authors presented a generalized Nash equilibrium model with stochastic demand to analyze competition among organizations at demand points for medical supplies. In [30], Nagurney and Salarpour introduced a two-stage stochastic game theory model in order to examine the behavior of national governments during Covid-19 pandemic, and their competition for essential medical supplies in both the preparation and response phases. All the problems presented in [28–30] were solved as variational inequalities, using the concept of variational equilibrium. We remark that our model differs from the treatment in [26] as we develop a variational inequality approach. In addition, although the problems introduced in [28–30] have similarities, they are all restricted to the case of discrete probability distribution, whereas our model is valid also for general probability distribution. This poses challenges for both theory and computations.

Recently, two-stage stochastic variational inequalities have been introduced to model cases where one looks for a decision vector before the real situation is known, and a new one after the scenario has been realized. In [10], the authors formulated the two-stage stochastic variational inequality as a two-stage stochastic programming problem with recourse. In [23], Li and Zhang studied the transformation of a general two-stage stochastic programming problem to a two-stage stochastic variational inequality. In [14], the authors presented an evacuation model where a population had to be evacuated from crisis areas to shelters, and, due to the uncertainty in the size of the population to be evacuated, a two-stage stochastic variational inequality model was given. In [32], Rockafellar and Wets discussed the multistage stochastic variational inequality. In [33], the authors developed progressive hedging methods for solving multistage convex stochastic programming, see also [34].

In [16], Gwinner and Raciti studied the random variational inequality and general random equilibrium problems. In particular, they worked on a class of linear random variational inequalities on random sets, with results on measurability, existence and uniqueness in a Hilbert space. Furthermore, they provided an approximation procedure in a special case. Then, in [17], the same authors carried out the theory of random variational inequalities to study a class of random equilibrium problems on networks in the linear case, and in [18] they studied the application to nonlinear random traffic equilibrium problem. A valuable additional contribution of the same authors is the book in [19]. In [11], the authors formulated the multicriteria spatial price network equilibrium problem as a random variational inequality, in which the consumers weight, using random fluctuations, transportation cost and the transportation time associated with the shipment of a given item. In [7, 8], the authors applied a general random traffic equilibrium problem, featuring the random Wardrop equilibrium distribution using random variational inequality. In [22], Jadamba and Raciti explored stochastic Nash equilibrium problems using monotone variational inequalities in probabilistic Lebesgue spaces. Their results are applied to a class of oligopolistic market equilibrium problems.

Inspired by the above works, in this paper, we provide a variational inequality formulation of the two-stage stochastic optimization problem describing the competition of healthcare institutions in case an emergency happens. The main contributions of our work are:

- Modeling a medical supply network that involves warehouses and hospitals with multiple medical items and multiple transportation modes.
- Providing a two-stage stochastic programming model based on disaster scenarios that considers the unmet demand at the first stage and the consequent penalty at the second stage.
- Deriving a variational inequality formulation in both the discrete and general probability cases.
- Characterizing the second-stage equilibrium, in the case of general probability distribution, by means of infinite-dimensional Lagrange duality.
- Testing the equilibrium model with numerical illustrations with realistic data.

An analysis of the Lagrange multipliers is also performed and, hence, this paper adds to the literature on the study of marginal utilities in the more challenging setting of stochastic programming problems.

This paper is organized as follows. In Sect. 2, we introduce the two-stage stochastic model for the medical supply competition. In Sect. 3, we present the stochastic Nash equilibrium concept underlying our model and the equivalent variational inequality formulation. The cases of discrete and general probability distribution are discussed. In Sect. 4, we recall some infinite-dimensional duality tools, and, in Sect. 5, we present an alternative formulation of the second-stage problem. The progressive hedging algorithm is then applied to some numerical examples in Sect. 6. We summarize our results and draw our conclusions in Sect. 7.

**Fig. 1:**
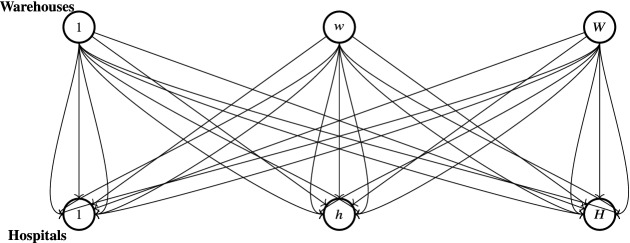
The Network representation

## The Two-Stage Stochastic Model

In this section, we present our two-stage stochastic model for the medical supply competition, see also [14]. Let ${\mathcal {W}}$ be the set of warehouses, with typical warehouse denoted by *w*; let ${\mathcal {H}}$ be the set of hospitals, with typical hospital denoted by *h*; let ${\mathcal {K}}$ be the set of medical supply type, with typical type denoted by *k*, and let ${\mathcal {M}}$ be the set of transportation modes, with typical mode denoted by *m*. We consider a network model as in Fig. 1. The links between the nodes of the network represent all the possible connections between the warehouses and the hospitals. Multiple links between each warehouse and each hospital describe the possibility of alternative modes of transportation. We note that the suitable transportation mode is often connected to the distance between supply and demand locations. Thus, for long distances airplanes are preferred to transportation by truck or train. The choice of the transportation mode may also depend on the type of medical item or on the severity of the emergency.

Let $x^{k}_{wh}$ be the amount of medical item of type *k* from warehouse *w* to hospital *h*, and let $\rho _{w}^{k}$ be the unitary price of medical item *k* at warehouse *w*. Let $x_{wh}$ denote the total amount delivered from warehouse *w* to hospital *h*, where

$$\begin{aligned} x_{wh}=\sum _{k\in {\mathcal {K}}} x_{wh}^k. \end{aligned}$$

We further group the $x_{wh}$ into the $WH-$dimensional column vector *x*.

Moreover, we introduce the transportation time $t_{wh}^m$ from warehouse *w* to hospital *h* with mode *m* and assume that it depends on the total amount *x*, namely, $t_{wh}^m=t_{wh}^m(x)$. We consider two stages, where one corresponds to the preparedness phase and the other represents the response phase. In the first phase, each demand location, namely the hospital, looks for minimizing the purchasing cost of medical items and the transportation time from the first stage; in the second one, a recourse decision process is developed to optimize the transportation costs from the second stage, in response to each disaster scenario. Let $(\Omega ,{\mathcal {F}},P)$ be a probability space, where the random parameter $\omega \in \Omega$ represents the typical disaster scenario. For each $\omega \in \Omega$, we denote by $\xi :\Omega \rightarrow {\mathbb {R}}^{WHK+HK}$ a finite-dimensional random vector and by ${\mathbb {E}}_{\xi }$ the mathematical expectation with respect to $\xi$. In order to formulate the two-stage stochastic model, we introduce two types of decision variables. The first-stage decision variable $x^{k}_{wh}$ is used to represent the quantity of medical supplies of type *k* from warehouse *w* to hospital *h* in stage one. The second-stage decision variables are $y^{k}_{wh}(\omega )$ and $z_h^k(\omega )$. The variable $y^{k}_{wh}(\omega )$ represents the quantity of medical supplies of type *k* to be delivered from warehouse *w* to hospital *h* under scenario $\omega$. The variable $z_h^k(\omega )$ is the unfulfilled demand at hospital *h* of medical supply item *k* under scenario $\omega$. The amount of unfulfilled demand $z_h^k(\omega )$ is penalized by the penalty function $\pi _h^k=\pi _h^k(\omega ,z_h^k(\omega ))$. We note that $x^k_{wh}$ is chosen before a realization of $\xi$ is revealed and later $y^k_{wh}(\omega )$ and $z^k_{h}(\omega )$ are selected with known realization. We set $y_{wh}(\omega )=\sum _{k\in {\mathcal {K}}}y_{wh}^k(\omega )$. We further group the $y_{wh}(\omega )$ into the $WH-$dimensional column vector $y(\omega )$. Finally, we introduce the transportation cost $c_{wh}^m$ from warehouse *w* to hospital *h* with mode *m* and assume that it depends on the total amount $y(\omega )$, namely, $c_{wh}^m=c_{wh}^m(\omega ,y(\omega ))$. Table 1 summarizes the relevant notations used in the model formulation.

**Table 1:** The notation for the two-stage stochastic model

Symbols	Definitions
${\mathcal {W}}$	Set of warehouses, with typical warehouse denoted by *w*, $card({\mathcal {W}})=W$
${\mathcal {H}}$	Set of hospitals, with typical hospital denoted by *h*, $card({\mathcal {H}})=H$
${\mathcal {K}}$	Set of different medical items, with typical item denoted by *k*, $card({\mathcal {K}})=K$
${\mathcal {M}}$	Set of transportation modes, with typical mode denoted by *m*, $card({\mathcal {M}})=M$
$d^{k}_h$	Demand of medical item *k* of hospital *h* in stage one
$d^{k}_h(\omega )$	Demand of medical item *k* of hospital *h* in stage two under scenario $\omega$
$x^{k}_{wh}$	Amount of medical item *k* from warehouse *w* to hospital *h* in stage one
$x_{wh}$	Amount of medical items delivered from warehouse *w* to hospital *h* in stage one
*x*	Amount of total medical items from all warehouses to all hospitals in stage one
$e_k$	Maximum amount available of medical item *k*
$\rho ^{k}_{w}$	Unitary price of medical item *k* at warehouse *w*
$t_{wh}^m(x)$	Transportation time from warehouse *w* to hospital *h* with mode *m*
$y_{wh}(\omega )$	Amount of medical items to be delivered from warehouse *w* to hospital *h* in stage two under scenario $\omega$
$y(\omega )$	Amount of medical items to be delivered from all warehouses to all hospitals in stage two under scenario $\omega$
$z_h^k(\omega )$	Amount of unfulfilled demand at hospital *h* of medical supply item *k* under scenario $\omega$
$c_{wh}^m(\omega ,y(\omega ))$	Transportation cost from warehouse *w* to hospital *h* with mode *m* under scenario $\omega$
$\pi _{h}^k(\omega ,z_{h}^k(\omega ))$	Penalty for unfulfilled demand at hospital *h* of medical supply item *k* under scenario $\omega$

We aim at obtaining an efficient plan of medical item procurement of each demand location in the first stage by the evaluation of adaptive plans in the second stage. Thus, for each hospital, we minimize the purchasing cost and the transportation time of the first stage with the expected overall costs and the penalty for the prior plan. For each hospital, a two-stage procurement planning model in a random environment is formulated. We first present the hospital’s problem as a two-stage stochastic programming problem and then define the stochastic Nash equilibrium describing the competition of all hospitals.

For each hospital *h*, the first-stage problem is given by

1 $$\begin{aligned}&\min \sum _{w\in {\mathcal {W}}}\Bigg (\sum _{k \in {\mathcal {K}}}\rho ^{k}_{w}x^{k}_{wh} +\sum _{m\in {\mathcal {M}}} t_{wh}^m({x})\Bigg )+ {\mathbb {E}}_{\xi }(\Phi _h(x,\xi (\omega ))) \end{aligned}$$

2 $$\begin{aligned}&\sum _{w\in {\mathcal {W}}}x^{k}_{wh}{\ge } d_h^k,\, \forall k\in {\mathcal {K}}, \end{aligned}$$

3 $$\begin{aligned}&\sum _{w\in {\mathcal {W}}}x^{k}_{wh}\le e_k, \, \forall k\in {\mathcal {K}}, \end{aligned}$$

4 $$\begin{aligned}&x^{k}_{wh}\ge 0,\, \forall w\in {\mathcal {W}},\, \forall k\in {\mathcal {K}}. \end{aligned}$$

The objective function (1) minimizes the sum of the purchasing cost for early supply plan, the transportation time, and the expected value of the second stage solution, with respect to disaster scenario, $\Phi _h(x,\xi (\omega ))$. Constraint (2) states that hospital *h* receives at least the needed amount of medical items; constraint (3) is the maximum availability constraint of each medical supply type *k*; constraint (4) is the non-negativity requirement on variables. In order to ensure that the constraint set is nonempty, we require that $d_h^k\le e_k, \forall h,k$.

For a given realization $\omega \in \Omega$, $\Phi _h(x,\xi (\omega ))$ is given by

5 $$\begin{aligned}&\Phi _h(x,\xi (\omega ))=\min \sum _{w\in {\mathcal {W}}}\sum _{m\in {\mathcal {M}}}c_{wh}^m(\omega ,{y}(\omega ))+\sum _{k\in {\mathcal {K}}} \pi _{h}^k(\omega , z^k_{h}(\omega )) \end{aligned}$$

6 $$\begin{aligned}&\text {subject to}\nonumber \\&\sum _{w\in {\mathcal {W}}} y^{k}_{wh}(\omega )+z^{k}_{h}(\omega ) {\ge } d_h^k(\omega ),\,\forall k\in {\mathcal {K}}, {P-a.s. }, \end{aligned}$$

7 $$\begin{aligned}&\sum _{w\in {\mathcal {W}}}y^{k}_{wh}(\omega )+\sum _{w\in {\mathcal {W}}}x^{k}_{wh}\le e_k(\omega ),\, \forall k\in {\mathcal {K}}, {P-a.s. }, \end{aligned}$$

8 $$\begin{aligned}&y^{k}_{wh}(\omega )\ge 0,z^k_{h}(\omega )\ge 0, z^k_{h}(\omega )\le \alpha \,d_h^k, \forall w\in {\mathcal {W}},\, \forall k \in {\mathcal {K}}, {P-a.s. } \end{aligned}$$

Thus, $\Phi _h(x,\xi (\omega ))$ is the optimal value of the second-stage problem (5)–(8) associated with hospital *h*, where the constraints hold almost surely (P-a.s.). We remark that $\Phi _h(x,\xi (\omega ))$ depends on *x* via constraint (7). The objective function (5) minimizes the total cost and the penalty for unmet demand at the second stage. Constraint (6) states that the supply at the second stage plus the unmet demand should be at least as much as the demand at the second stage. Constraint (7) is the maximum availability constraint of each medical supply of type *k*. We emphasize that the connection between stage-wise decision variables *x* and *y* is captured by constraint (7). It is the linking factor between the first and second stage and communicates the first-stage decisions to the second one. Constraint (8) is the non-negativity requirement on variables. We also assume that $z^k_{h}(\omega )\le \alpha \,d_h^k$, $\alpha \in ]0,1]$, ${P-a.s. }$, namely, the unmet demand cannot exceed a fixed percentage of the first-stage demand.

In order to ensure that the constraint set of the second stage is nonempty, it suffices to require that

$$\begin{aligned} d_h^k(\omega )\le \min \bigg \{e_k(\omega ),\frac{d_h^k}{\alpha }\bigg \},\, \forall h,k, {P-a.s. } \end{aligned}$$

We assume that:

- $t_{wh}^m(\cdot )$ is continuously differentiable and convex for all *w*, *h*, *k*;
- $c_{wh}^m(\omega ,\cdot )$, $\pi _h^k(\omega ,\cdot )$, a.e. in $\Omega$, are continuously differentiable and convex for all *w*, *h*, *k*, *m*;
- for each $u\in {\mathbb {R}}^{WH}$, $c_{wh}^m(\cdot ,u)$ is measurable with respect to the random parameter in $\Omega$ for all *w*, *h*, *m*;
- for each $v\in {\mathbb {R}}^{HK}$, $\pi _{h}^k(\cdot ,v)$ is measurable with respect to the random parameter in $\Omega$ for all *h*, *k*;
- $\frac{{\partial }c_{wh}^m(\omega ,y(\omega ))}{\partial y^k_{wh}}$, $\frac{\partial \pi _h^k(\omega ,z_h^{k}(\omega ))}{\partial z^k_{h} }$ are measurable in $\omega$ and continuous in *y* and *z*;
- $y_{wh}^k:\Omega \rightarrow {\mathbb {R}}$ and $z_{h}^k:\Omega \rightarrow {\mathbb {R}}$ are measurable mappings for all *w*, *h*, *k*;
- $d_{h}^k:\Omega \rightarrow {\mathbb {R}}$ is a measurable mapping for all *h* and all *k*.

The two-stage problem of hospital *h* can be also formulated as the unique large-scale problem:

9 $$\begin{aligned}&\min \sum _{w\in {\mathcal {W}}}\Bigg (\sum _{k \in {\mathcal {K}}}\rho ^{k}_{wh}x^{k}_{wh} +\sum _{m\in {\mathcal {M}}} t_{wh}^m({x})\Bigg )+ {\mathbb {E}}_{\xi }(\Phi _h(x,\xi (\omega ))) \end{aligned}$$

10 $$\begin{aligned}&\text {subject to}\nonumber \\&\sum _{w\in {\mathcal {W}}}x^{k}_{wh}{\ge } d_h^k,\ \forall k\in {\mathcal {K}},{P-a.s. }, \end{aligned}$$

11 $$\begin{aligned}&\sum _{w\in {\mathcal {W}}}x^{k}_{wh}\le e_k,\,\forall k\in {\mathcal {K}}, {P-a.s. }, \end{aligned}$$

12 $$\begin{aligned}&\sum _{w\in {\mathcal {W}}} y^{k}_{wh}(\omega )+z^k_{h} (\omega ){\ge } d_h^k(\omega ),\,\forall k\in {\mathcal {K}}, {P-a.s. }, \end{aligned}$$

13 $$\begin{aligned}&\sum _{w\in {\mathcal {W}}}y^{k}_{wh}(\omega )+\sum _{w\in {\mathcal {W}}}x^{k}_{wh}\le e_k(\omega ),\, \forall k\in {\mathcal {K}}, {P-a.s. }, \end{aligned}$$

14 $$\begin{aligned}&x^{k}_{wh}\ge 0,\, \forall w\in {\mathcal {W}},\, \forall k\in {\mathcal {K}}, {P-a.s. }, \end{aligned}$$

15 $$\begin{aligned}&y^{k}_{wh}(\omega )\ge 0,\, \forall w\in {\mathcal {W}},\, \forall k \in {\mathcal {K}}, {P-a.s. }, \end{aligned}$$

16 $$\begin{aligned}&z^k_{h}(\omega ) \ge 0,\, \forall k \in {\mathcal {K}}, {P-a.s. }, \end{aligned}$$

17 $$\begin{aligned}&z^k_{h}(\omega )\le \alpha \,d_h^k,\, \forall k \in {\mathcal {K}}, {P-a.s. } \end{aligned}$$

## Stochastic Nash Equilibrium Problem

In this section, we present the equilibrium concept underlying our model and the equivalent variational inequality formulation. Both the cases of discrete and general probability distribution are discussed.

Each hospital minimizes the deterministic costs and the expected costs for all the scenarios. Then, the hospitals simultaneously solve their own optimization problems and reach a stable state governed by the Nash equilibrium concept.

We define the sets:

$$\begin{aligned}&S_h=\Big \{x_h=(x_{wh})_{w}\in {\mathbb {R}}^{W}: (10)-(11), (14) \text { hold} \Big \},\\&T_h=\Big \{(y_h,z_h)=(y_{wh}(\omega ),z_{hk}(\omega ))_{w,k}\in {\mathbb {R}}^{W+K}: (12)-(13), (15)-(17) \text { hold, } {P-a.s. } \Big \},\\&S=\prod _h S_h,\\&T=\prod _h T_h. \end{aligned}$$

We will refer to the objective function (9) as the function ${\mathbb {J}}_h(\omega ,x,y(\omega ),z(\omega ))$, namely

$$\begin{aligned}&{\mathbb {J}}_h(\omega ,x,y(\omega ),z(\omega ))= \sum _{w\in {\mathcal {W}}}\Bigg (\sum _{k \in {\mathcal {K}}}\rho ^{k}_{wh}x^{k}_{wh} +\sum _{m\in {\mathcal {M}}} t_{wh}^m({x})\Bigg )+ {\mathbb {E}}_{\xi }(\Phi _h(x,\xi (\omega ))). \end{aligned}$$

### Definition 3.1

A vector of medical items $(x^*,y^*,z^*) \in S\times T$ is a stochastic Nash equilibrium if for each $h\in {\mathcal {H}}$

$$\begin{aligned}&{\mathbb {J}}_h(\omega ,x_h^*,y_h^*(\omega ),z_h^*(\omega ),x_{-h}^*,y_{-h}^* (\omega ),z_{-h}^*(\omega ))\\&\quad \le {\mathbb {J}}_h(\omega ,x_h,y_h(\omega ),z_h(\omega ),x^*_{-h},y_{-h}^*(\omega ),z_{-h}^*(\omega )),\\&\forall (x_h,y_h(\omega ),z_h(\omega ))\in S_h\times T_h, \text { P-a.s. }, \end{aligned}$$

where $x_{-h},y_{-h}(\omega ),z_{-h}(\omega )$ denotes the amount of medical items and the unmet demands of all hospitals except for *h*.

According to the above definition, a Nash equilibrium is established if no hospital can unilaterally improve upon his profit by choosing an alternative medical item flow pattern, given other hospitals’ decision strategies.

### Discrete Probability Distribution

If the random parameter $\omega \in \Omega$ follows a discrete distribution with finite support $\Omega =\{\omega _1,\ldots ,\omega _r\}$ and probabilities $p(\omega _r)$ associated with each realization $\omega _r$, $r\in {\mathcal {R}}=\{1,\ldots ,R\}$, then the objective function (9) for $h\in {\mathcal {H}}$ becomes

18 $$\begin{aligned}&{\mathbb {J}}_h(\omega ,x,y(\omega ),z(\omega ))=\sum _{w\in {\mathcal {W}}}\Bigg (\sum _{k \in {\mathcal {K}}}\rho ^{k}_{wh}x^{k}_{wh} +\sum _{m\in {\mathcal {M}}} t_{wh}^m({x})\Bigg ) \nonumber \\&+\sum _{r\in {\mathcal {R}}} p(\omega _r)\Bigg ( \sum _{w\in {\mathcal {W}}}\sum _{m\in {\mathcal {M}}}c_{wh}^m(\omega _r,{y}(\omega _r))+\sum _{k\in {\mathcal {K}}}\pi _{h}^k( \omega _r,z^k_{h}(\omega _r))\Bigg ), \end{aligned}$$

and the cost minimization problem for $h\in {\mathcal {H}}$ is given by

19 $$\begin{aligned}&\min {\mathbb {J}}_h(\omega , x,y(\omega ),z(\omega )) \end{aligned}$$

20 $$\begin{aligned}&\sum _{w\in {\mathcal {W}}}x^{k}_{wh}{\ge } d_h^k,\ \forall k\in {\mathcal {K}}, \end{aligned}$$

21 $$\begin{aligned}&\sum _{w\in {\mathcal {W}}}x^{k}_{wh}\le e_k,\,\forall k\in {\mathcal {K}}, \end{aligned}$$

22 $$\begin{aligned}&\sum _{w\in {\mathcal {W}}} y^{k}_{wh}(\omega _r)+z^k_{h}(\omega _r){\ge } d_h^k(\omega _r),\,\forall k\in {\mathcal {K}}, \forall r\in {\mathcal {R}}, \end{aligned}$$

23 $$\begin{aligned}&\sum _{w\in {\mathcal {W}}}y^{k}_{wh}(\omega _r)+\sum _{w\in {\mathcal {W}}}x^{k}_{wh}\le e_k(\omega _r),\, \forall k\in {\mathcal {K}}, \forall r\in {\mathcal {R}}, \end{aligned}$$

24 $$\begin{aligned}&x^{k}_{wh}\ge 0,\, \forall w\in {\mathcal {W}},\, \forall k\in {\mathcal {K}}, \end{aligned}$$

25 $$\begin{aligned}&y^{k}_{wh}(\omega _r)\ge 0,\, \forall w\in {\mathcal {W}},\, \forall k \in {\mathcal {K}}, \forall r\in {\mathcal {R}}, \end{aligned}$$

26 $$\begin{aligned}&z^k_{h}(\omega _r) \ge 0,\, \forall k \in {\mathcal {K}}, \forall r\in {\mathcal {R}}, \end{aligned}$$

27 $$\begin{aligned}&z^k_{h}(\omega _r)\le \alpha \,d_h^k,\, \forall k \in {\mathcal {K}}, \forall r\in {\mathcal {R}}. \end{aligned}$$

It is well known that a Nash equilibrium can be characterized as a solution to a variational inequality problem (see [20, 27] for theory and applications on variational inequalities). Thus, the competition among hospitals under the Nash criterion is described by the following variational inequality:

28 $$\begin{aligned}&\sum _{w\in {\mathcal {W}}}\sum _{h\in {\mathcal {H}}}\sum _{k \in {\mathcal {K}}}\Bigg (\rho ^{k}_{wh} +\sum _{m\in {\mathcal {M}}}\frac{{\partial } t_{wh}^m(x^*)}{{\partial }x_{wh}^k}\Bigg )\times (x^k_{wh}-x^{*k}_{wh})\nonumber \\&\qquad +\sum _{r\in {\mathcal {R}}} p(\omega _r) \sum _{w\in {\mathcal {W}}}\sum _{h\in {\mathcal {H}}} \sum _{k \in {\mathcal {K}}} \Bigg (\sum _{m\in {\mathcal {M}}}\frac{{\partial }c_{wh}^m(\omega _r,y^*(\omega _r))}{{\partial }y^k_{wh}}\Bigg )\times (y_{wh}^{k}(\omega _r) -y^{*k}_{wh}(\omega _r))\nonumber \\&\qquad +\sum _{r\in {\mathcal {R}}} p(\omega _r) \sum _{h\in {\mathcal {H}}} \sum _{k \in {\mathcal {K}}} \frac{{\partial }\pi _h^k(\omega _r,z_h^{*k}(\omega _r))}{\partial z_h^k}\times (z_{h}^{k}(\omega _r) -z^{*k}_{h}(\omega _r))\ge 0,\nonumber \\&\forall (x,y(\omega _r),z(\omega _r))\in S\times T, \forall r\in {\mathcal {R}}. \end{aligned}$$

We note that the set *S*, and the set *T*, $\forall r\in {\mathcal {R}}$, are nonempty, compact and convex, and the operator entering (28) is continuous. Therefore, a solution to the above problem exists from the standard theory of variational inequalities [20].

### General Probability Distribution

In the case of a general probability space $(\Omega ,{\mathcal {F}},P)$, studying the optimality conditions can be very hard, as one should state the order of the decision process explicitly. For this reason, we choose as our functional setting a Hilbert space and assume that $y\in L^2(\Omega ,P, {\mathbb {R}}^{WH})$, $z\in L^2(\Omega ,P, {\mathbb {R}}^{HK})$, $d\in L^2(\Omega ,P, {\mathbb {R}}^{HK})$. $L^2(\Omega ,P, {\mathbb {R}}^{WH})$ denotes the class of ${\mathbb {R}}^{WH}$-valued functions defined in $\Omega$, that are square integrable with respect to the probability measure *P*.

Analogous meaning has the space $L^2(\Omega ,P, {\mathbb {R}}^{HK})$.

Moreover, we require the following growth conditions, $\forall m,w,h,k$:

29 $$\begin{aligned} \Big |c_{wh}^m(\omega , y)\Big |&\le \beta ^{1m}_{wh}(\omega )(1+\Vert y\Vert ), \forall y\in {\mathbb {R}}^{WH}, \,\nonumber \\ \Big |\pi _h^k(\omega , z)\Big |&\le \beta ^{2k}_{h}(\omega )(1+\Vert z\Vert ), \forall z\in {\mathbb {R}}^{HK}, \text { P-a.s. }, \end{aligned}$$

30 $$\begin{aligned} \Bigg |\frac{{\partial }c_{wh}^m(\omega , y)}{{\partial }y^k_{wh} }\Bigg |&\le \beta _{wh}^{3m}(\omega )(1+\Vert y\Vert ), \forall y\in {\mathbb {R}}^{WH},\,\nonumber \\ \Bigg |\frac{{\partial }\pi _h^k(\omega ,z_h)}{\partial z_h^k}\Bigg |&\le \beta _h^{4k}(\omega )(1+\Vert z\Vert ), \forall z\in {\mathbb {R}}^{HK}, \text { P-a.s. }, \end{aligned}$$

where $\beta ^{1m}_{wh}, \beta ^{2k}_{h}, \beta ^{3m}_{wh}, \beta ^{4k}_{h}$ are nonnegative functions of $L^{\infty }(\Omega )$.

#### Theorem 3.1

Under assumptions $$a)-g) $$ and conditions (29)–(30), a vector $$(x^*,y^*,z^*) \in S\times T$$ is an optimal solution of the medical supply problem if and only if it is a solution of the following variational inequality:

31 $$\begin{aligned}&\sum _{w\in {\mathcal {W}}}\sum _{h\in {\mathcal {H}}}\sum _{k \in {\mathcal {K}}}\Bigg (\rho ^{k}_{wh} +\sum _{m\in {\mathcal {M}}}\frac{{\partial } t_{wh}^m(x^*)}{{\partial }x_{wh}^k}\Bigg )\times (x^k_{wh}-x^{*k}_{wh})\nonumber \\&+\sum _{h\in {\mathcal {H}}} \sum _{k \in {\mathcal {K}}}\int _{\Omega }\Bigg [ \sum _{w\in {\mathcal {W}}}\Bigg (\sum _{m\in {\mathcal {M}}}\frac{{\partial }c_{wh}^m(\omega ,y^*(\omega ))}{{\partial }y^k_{wh} }\Bigg )\times (y_{wh}^{k}(\omega ) -y^{*k}_{wh}(\omega ))\nonumber \\&+ \frac{{\partial }\pi _h^k(\omega ,z_h^{*k}(\omega ))}{\partial z_h^k}\times (z_{h}^{k}(\omega ) -z^{*k}_{h}(\omega )))\Bigg ]dP(\omega ) \ge 0,\quad \forall (x,y,z)\in S\times T . \end{aligned}$$

#### Proof

The proof procceds as in [3]. $\square$ $\square$

To ensure the existence of solutions, we may apply the results in [25]. We first recall some definitions.

Let *E* be a reflexive Banach space with dual space $E^*$ and $K\subset E$ a closed convex set.

#### Definition 3.2

A mapping $A : K \mapsto E^*$ is called pseudomonotone in the sense of Brezis if and only if

- for each sequence $u_n$ weakly converging to *u* in *K* and such that $\limsup _n \langle Au_n, u_n -u\rangle \le 0$ it results $\liminf _n \langle Au_n,u_n-v\ge \langle Au,u-v\rangle , \quad \forall v\in K;$
- for each $v\in K$ the function $u\mapsto \langle Au,u-v\rangle$ is lower bounded on the bounded subsets of *K*.

#### Definition 3.3

A mapping $A : K\mapsto E^*$ is hemicontinuous in the sense of Fan if and only if for all $v\in K$ the function $u\mapsto \langle Au,u-v\rangle$ is weakly lower semicontinuous on *K*.

#### Definition 3.4

The map $A: K \rightarrow E^*$ is said to be lower hemicontinuous along line segments, if and only if the function:

$$\begin{aligned} \xi \mapsto \langle A \xi ,u-v \rangle \end{aligned}$$

is lower semicontinuous for all $u,v \in K$ on the line segments [*u*, *v*].

#### Definition 3.5

The map $A: K \rightarrow E^*$ is said to be pseudomonotone in the sense of Karamardian if and only if for all $u,v \in K$

$$\begin{aligned} \langle A v,u-v \rangle \ge 0 \rightarrow \langle Au, u-v \rangle \ge 0. \end{aligned}$$

#### Theorem 3.2

Let us assume that the map $A:K\mapsto E^*$ be B-pseudomonotone or F-hemicontinuous and there exist $u_0\in K$ and $R >\Vert u_0\Vert$ such that

32 $$\begin{aligned}&\langle A v, v-u_0\rangle \ge 0,\quad \forall v\in K\cap \{v \in E: \Vert v\Vert =R\}. \end{aligned}$$

Then, the variational inequality $\langle A u,v-u\rangle , \forall v\in K$ admits solutions.

#### Theorem 3.3

Let $A:K\mapsto E^*$ be a K-pseudomonotone map which is lower hemicontinuous along line segments. Let us assume that condition (32) holds true. Then, variational inequality $\langle A u,v-u\rangle , \forall v\in K$ admits solutions.

We recall that condition (32) is satisfied if the coercivity condition is verified:

33 $$\begin{aligned} {\mathop {\mathop {\lim }_{\Vert u\Vert \rightarrow \infty }}\limits _{u\in K}} \frac{\langle A u,u-u_{0}\rangle }{\Vert u\Vert }=+\infty . \end{aligned}$$

We can apply Theorem 3.2 and Theorem 3.3, assuming that the operator of the variational inequality is B-pseudomonotone or F-hemicontinuous and (32) or (33) holds true, or assuming that it is K-pseudomonotone, conditions (29)–(30) are verified, and (32) or (33) holds true. We also recall that condition (30) is sufficient to guarantee that the operator is lower hemicontinuous along line segments (see [15]).

## Duality Theory

We now present some infinite-dimensional Lagrange duality results as in [4–6, 9]. For reader’s convenience, we first recall some typical concepts in duality theory [21]. Let *X* denote a real normed space and $X^*$ the topological dual of all continuous linear functionals on *X*. Given *C*, a nonempty subset of *X*, and an element $x\in X$, the set

$$\begin{aligned} T_C(x):= & {} \Big \{h\in X: h=\lim _{n\rightarrow \infty } \lambda _n(x_n-x), \lambda _n\in {\mathbb {R}}, \lambda _n>0 \,\forall n\in {\mathbb {N}},\\&\qquad x_n\in C \,\forall n\in {\mathbb {N}},\,\lim _{n\rightarrow \infty } x_n=x\Big \} \end{aligned}$$

is called the contingent cone to *C* at *x*. Of course, if $T_C(x)\ne \emptyset$, then *x* belongs to the closure of *C*, denoted by $\text {cl } C$. If *C* is convex, then [21]

$$\begin{aligned} T_C(x)=\text {cl cone} (C-\{x\}),\text {where cone}(C):\,=\{\lambda x:x\in C, \lambda \in {\mathbb {R}},\lambda \ge 0\}. \end{aligned}$$

We now present the statement of Theorem 3.2 in [24]. Let *X* be a real normed space real and *S* be a nonempty subset of *X*; let $(Y,\Vert \cdot \Vert )$ be a real normed space, partially ordered by a convex cone *C*. Let $f : S \rightarrow {\mathbb {R}}$ and $g : S \rightarrow Y$ be two convex functions. Let us consider the primal problem

34 $$\begin{aligned} \min _{x\in K} f(x), \quad K:=\{x\in S: g(x)\in -C\}, \end{aligned}$$

and the dual problem

35 $$\begin{aligned} \max _{u\in C^*}\inf _{x\in S}\{f(x)+\langle u,g(x) \rangle \},\quad C^*:=\Big \{u\in Y^*: \langle u,v \rangle \ge 0, \forall v\in C\Big \}, \end{aligned}$$

where $C^*$ is the dual cone of *C*.

We say that *Assumption* *S* is fulfilled at a point $x_0 \in K$ if and only if it results:

$$\begin{aligned} T_{\widetilde{M}} (f(x_0), 0_Y) \cap ]-\infty , 0[ \times \{0_Y\}=\emptyset , \end{aligned}$$

where

$$\begin{aligned} \widetilde{M}:=\Big \{(f(x)-f(x_0)+\gamma , g(x)+v):x\in S\setminus K,\alpha \ge 0,v,y\in C\Big \}. \end{aligned}$$

Then, in [24] the following theorem is proved.

### Theorem 4.1

Under the above assumptions, if problem (34) is solvable and Assumption *S* is fulfilled at the extremal solution $x_0\in K$, then also problem (35) is solvable, the extreme values of both problems are equal and, denoted by $\overline{u}$ the optimal solution of (35), it results that $\langle \overline{u}, g(x_0)\rangle =0$.

The following result entitles us to characterize a solution of problem (34) as a saddle point of the Lagrange function [4].

### Theorem 4.2

Let us assume that assumptions of Theorem 4.1 be satisfied. Then, $x_0\in K$ is a minimal solution to problem (34) if and only if there exists $\overline{u} \in C^*$ such that $(x_0,\overline{u})$ is a saddle point of the Lagrange function, namely,

$$\begin{aligned} {\mathcal {L}}(x_0,u)\le {\mathcal {L}}(x_0,\overline{u})\le {\mathcal {L}}(x,\overline{u}), \forall x\in S, u\in C^*,\quad \langle \overline{u}, g(x_0)\rangle =0. \end{aligned}$$

We now apply the duality framework in [4–6] to the second-stage problem (5)–(8). First, we note problem (5)–(8) is equivalent to a variational inequality, see [3].

### Theorem 4.3

The vector $(y^*_h,z^*_h) \in T_h$, for all $h\in {\mathcal {H}}$, is an optimal solution of the second-stage problem (5)–(8) if and only if $(y^*_h,z^*_h)\in T_h$ solves the variational inequality

36 $$\begin{aligned}&\sum _{k \in {\mathcal {K}}} \int _{\Omega }\Bigg [ \sum _{w\in {\mathcal {W}}}\Bigg (\sum _{m\in {\mathcal {M}}}\frac{{\partial }c_{wh}^m(\omega ,y^*(\omega ))}{{\partial }y^k_{wh} }\Bigg )\times (y_{wh}^{k}(\omega ) -y^{*k}_{wh}(\omega ))\nonumber \\&\qquad \qquad \quad + \frac{{\partial }\pi _h^k(\omega ,z_h^{*k}(\omega ))}{\partial z_h^k}\times (z_{h}^{k}(\omega ) -z^{*k}_{h}(\omega )))\Bigg ]dP(\omega ) \ge 0, \forall (y_h,z_h))\in T_h. \end{aligned}$$

Now, we give two preliminary results.

### Lemma 4.1

Let $(y^*_h,z^*_h) \in T_h$ be a solution to (36). Let us introduce, a.e. in $\Omega$,

$$\begin{aligned} \nu _{h}^{1}(\omega )&=\min \Bigg \{\sum _{m\in {\mathcal {M}}}\frac{{\partial }c_{wh}^m(\omega ,y^*(\omega ))}{{\partial }y^k_{wh} }:w\in {\mathcal {W}}, k\in {\mathcal {K}} \Bigg \},\,\\ \nu ^{2}_h(\omega )&=\min \Bigg \{\frac{{\partial }\pi _h^k(\omega ,z_h^{*k} (\omega ))}{\partial z_h^k}: k\in {\mathcal {K}}\Bigg \}\\ \Omega _{w}^{1k}&=\Bigg \{\omega \in \Omega : \sum _{m\in {\mathcal {M}}}\frac{{\partial }c_{wh}^m(\omega ,y^*(\omega ))}{{\partial }y^k_{wh}} =\nu _{h}^{1}(\omega )\Bigg \},w\in {\mathcal {W}}, k\in {\mathcal {K}} \\ \Omega _{w}^{2k}&=\Bigg \{\omega \in \Omega : \sum _{m\in {\mathcal {M}}}\frac{{\partial }c_{wh}^m(\omega ,y^*(\omega ))}{{\partial }y^k_{wh}}>\nu _{h}^{1}(\omega )\Bigg \}, w\in {\mathcal {W}}, k\in {\mathcal {K}} \\ \Omega ^{3k}&=\Bigg \{\omega \in \Omega :\frac{{\partial }\pi _h^k(\omega ,z_h^{*k}(\omega ))}{\partial z_h^k} =\nu ^{2}_h(\omega )\Bigg \}, k\in {\mathcal {K}},\, \\ \Omega ^{4k}&=\Bigg \{\omega \in \Omega : \frac{{\partial }\pi _h^k(\omega ,z_h^{*k}(\omega ))}{\partial z_h^k} >\nu ^{2}_h(\omega )\Bigg \}, k\in {\mathcal {K}}. \end{aligned}$$

Then,

37 $$\begin{aligned}&\omega \in \Omega _{w}^{1k}\Rightarrow y_{wh}^k(\omega )\ge 0, \omega \in \Omega _{w}^{2k}\Rightarrow y_{wh}^k(\omega )= 0, \end{aligned}$$

38 $$\begin{aligned}&\omega \in \Omega ^{3k}\Rightarrow z_{h}^k(\omega )\ge 0,\omega \in \Omega ^{4k}\Rightarrow z_{h}^k(\omega )= 0. \end{aligned}$$

Vice versa, if there exist two functions $\nu _{h}^{1},\nu ^{2}_h\in L^2(\Omega ,P,{\mathbb {R}})$ such that (37)–(38) hold, then $(y^*_h,z^*_h) \in T_h$ solves (36).

### Proof

We assume that $(y^*_h,z^*_h) \in T_h$ is a solution to (36). Following [3], we prove that if there exist $w_1,k_1,w_2,k_2$ such that

39 $$\begin{aligned} \sum _{m\in {\mathcal {M}}} \frac{{\partial }c_{w_1h}^m(\omega ,y^*(\omega ))}{{\partial }y^{k_1}_{w_1h}}<\sum _{m\in {\mathcal {M}}}\frac{{\partial }c_{w_{2}h}^m(\omega ,y^*(\omega ))}{{\partial }y^{k2}_{w_{2}h}}, \end{aligned}$$

then $y^{k2}_{w_{2}h}=0$. By contradiction, suppose that there exists a set $E\subseteq \Omega$, with positive measure, such that $y^{k2}_{w_{2}h}>0$, for all $\omega \in E$ and (39) holds. Let us set

$$\begin{aligned} y^{k}_{wh}=\left\{ \begin{array}{ll} y^{*k}_{wh} &{} \text {in } \Omega \setminus E,\\ y^{*k}_{wh} &{} \text {if } w\ne w_1,w_2, k\ne k_1,k_2, \text {in } E,\\ y^{*k_1}_{w_1h}+y^{*k_2}_{w_2h}&{} \text {if } w=w_1, k=k_1, \text {in } E,\\ 0&{} \text {if } w=w_2, k=k_2, \text {in } E, \end{array}\right. \end{aligned}$$

with $\sum _{w\in {\mathcal {W}}}x^{k}_{wh}{\ge } d_h^k$, $\sum _{w\in {\mathcal {W}}}x^{k}_{wh}\le e_k$, $x^{k}_{wh}\ge 0$,$\forall w\in {\mathcal {W}},\, \forall k\in {\mathcal {K}}$ and $z_h^{k}(\omega )=z_h^{*k}(\omega )$, $\forall k\in {\mathcal {K}}$. Variational inequality (36) becomes

$$\begin{aligned}&\sum _{k \in {\mathcal {K}}} \int _{\Omega \setminus E}\Bigg [ \sum _{w\in {\mathcal {W}}}\Bigg (\sum _{m\in {\mathcal {M}}}\frac{{\partial }c_{wh}^m(\omega ,y^*(\omega ))}{{\partial }y^k_{wh} }\Bigg )\times (y_{wh}^{k}(\omega ) -y^{*k}_{wh}(\omega ))\Bigg ]dP(\omega )\\&+ \sum _{\begin{array}{c} k \in {\mathcal {K}}\\ k\ne k_1,k_2 \end{array}} \int _{ E}\Bigg [ \sum _{\begin{array}{c} w\in {\mathcal {W}}\\ w\ne w_1,w_2 \end{array}}\Bigg (\sum _{m\in {\mathcal {M}}}\frac{{\partial }c_{wh}^m(\omega ,y^*(\omega ))}{{\partial }y^k_{wh} }\Bigg )\times (y_{wh}^{k}(\omega ) -y^{*k}_{wh}(\omega ))\Bigg ]dP(\omega )\\&+ \int _{ E}\Bigg (\sum _{m\in {\mathcal {M}}}\frac{{\partial }c_{w_1h}^m(\omega ,y^*(\omega ))}{{\partial }y^{k_1}_{w_1h} }\Bigg )\times (y_{w_1h}^{k_1}(\omega ) -y^{*k_1}_{w_1h}(\omega ))dP(\omega )\\&+ \int _{ E}\Bigg (\sum _{m\in {\mathcal {M}}}\frac{{\partial }c_{w_2h}^m(\omega ,y^*(\omega ))}{{\partial }y^{k_2}_{w_2h} }\Bigg )\times (y_{w_2h}^{k_2}(\omega ) -y^{*k_2}_{w_2h}(\omega ))dP(\omega )\\&= \int _{ E}\Bigg (\sum _{m\in {\mathcal {M}}}\frac{{\partial }c_{w_1h}^m(\omega ,y^*(\omega ))}{{\partial }y^{k_1}_{w_1h} }-\sum _{m\in {\mathcal {M}}}\frac{{\partial }c_{w_2h}^m(\omega ,y^*(\omega ))}{{\partial }y^{k_2}_{w_2h} }\Bigg )y_{w_2h}^{k_2}(\omega )dP(\omega )<0. \end{aligned}$$

This contradicts variational inequality (36). Thus, we have

$$\begin{aligned}&\sum _{m\in {\mathcal {M}}}\frac{{\partial }c_{wh}^m(\omega ,y^*(\omega ))}{{\partial }y^k_{wh}}=\nu _{h}^{1}(\omega ) \Rightarrow y_{wh}^{*k}(\omega )\ge 0, \\&\quad \sum _{m\in {\mathcal {M}}}\frac{{\partial }c_{wh}^m(\omega ,y^*(\omega ))}{{\partial }y^k_{wh}}>\nu _{h}^{1}(\omega ) \Rightarrow y_{wh}^{*k}(\omega )=0. \end{aligned}$$

Analogously, we find

$$\begin{aligned}&\frac{{\partial }\pi _h^k(\omega ,z_h^{*k}(\omega ))}{\partial z_h^k} =\nu ^{2}_h(\omega )\Rightarrow z_{h}^{*k}(\omega )\ge 0, \frac{{\partial }\pi _h^k(\omega ,z_h^{*k}(\omega ))}{\partial z_h^k} >\nu ^{2}_h(\omega ) \Rightarrow z_{h}^{*k}(\omega )=0. \end{aligned}$$

Now, we suppose that there exist two functions $\nu _{h}^{1},\nu ^{2}_h\in L^2(\Omega ,P,{\mathbb {R}})$ such that (37)–(38) hold. Variational inequality (36) becomes

$$\begin{aligned}&\sum _{k \in {\mathcal {K}}} \int _{\Omega }\Bigg [ \sum _{w\in {\mathcal {W}}}\Bigg (\sum _{m\in {\mathcal {M}}}\frac{{\partial }c_{wh}^m(\omega ,y^*(\omega ))}{{\partial }y^k_{wh} }\Bigg )\times (y_{wh}^{k}(\omega ) -y^{*k}_{wh}(\omega ))\\&\quad + \frac{{\partial }\pi _h^k(\omega ,z_h^{*k}(\omega ))}{\partial z_h^k}\times (z_{h}^{k}(\omega ) -z^{*k}_{h}(\omega )))\Bigg ]dP(\omega )\\&= \sum _{w \in {\mathcal {W}}} \sum _{k \in {\mathcal {K}}} \int _{\Omega ^{1k}_w} \nu _h^{1}(\omega )(y_{wh}^{k}(\omega ) -y^{*k}_{wh}(\omega ))dP(\omega )\\&\quad +\sum _{w \in {\mathcal {W}}} \sum _{k \in {\mathcal {K}}} \int _{\Omega ^{2k}_w} \sum _{w\in {\mathcal {W}}}\Bigg (\sum _{m\in {\mathcal {M}}}\frac{{\partial } c_{wh}^m(\omega ,y^*(\omega ))}{{\partial }y^k_{wh} }\Bigg )(y_{wh}^{k}(\omega ) -y^{*k}_{wh}(\omega ))dP(\omega ) \\&\quad +\sum _{w \in {\mathcal {W}}} \sum _{k \in {\mathcal {K}}} \int _{\Omega ^{3k}} \nu ^{2}_h(\omega )(z_{h}^{k}(\omega ) -z^{*k}_{h}(\omega ))dP(\omega )\\&\quad + \sum _{w \in {\mathcal {W}}} \sum _{k \in {\mathcal {K}}} \int _{\Omega ^{4k}}\sum _{w\in {\mathcal {W}}}\Bigg (\frac{{\partial }\pi _{h}^k(\omega ,z^{*k}_h(\omega ))}{{\partial }z^k_{h} }\Bigg )(z_{h}^{k}(\omega ) -z^{*k}_{h}(\omega ))dP(\omega )\\&\ge \sum _{w \in {\mathcal {W}}} \sum _{k \in {\mathcal {K}}} \int _{\Omega ^{1k}_w} \nu _h^{1}(\omega )(y_{wh}^{k}(\omega ) -y^{*k}_{wh}(\omega ))dP(\omega )\\&\quad + \sum _{w \in {\mathcal {W}}} \sum _{k \in {\mathcal {K}}} \int _{\Omega ^{2k}_w} \nu _h^{1}(\omega )(y_{wh}^{k}(\omega ) -y^{*k}_{wh}(\omega ))dP(\omega )\\&\quad +\sum _{w \in {\mathcal {W}}} \sum _{k \in {\mathcal {K}}} \int _{\Omega ^{3k}} \nu ^{2}_h(\omega )(z_{h}^{k}(\omega ) -z^{*k}_{h}(\omega ))dP(\omega )\\&\quad +\sum _{w \in {\mathcal {W}}} \sum _{k \in {\mathcal {K}}} \int _{\Omega ^{4k}} \nu ^{2}_h(\omega )(z_{h}^{k}(\omega ) -z^{*k}_{h}(\omega ))dP(\omega )=0. \end{aligned}$$

Therefore, variational inequality (36) is satisfied.$\square$

Now, we prove that *Assumption* *S* is verified.

### Theorem 4.4

Problem (36) verifies Assumption *S* at the optimal solution $(y^*_h,z^*_h) \in T_h$.

### Proof

We suppose that $(y^*_h,z^*_h) \in T_h$ is a solution to (36) and prove that *Assumption* *S* is verified at $(y^*_h,z^*_h) \in T_h$. We set $Y=L^2(\Omega ,P, {\mathbb {R}}^{WK})$, $Z=L^2(\Omega ,P, {\mathbb {R}}^{K})$ and prove that if $(l,\theta _Y,\theta _Y,\theta _Y,\theta _Z,\theta _Z )$ is such that

40 $$\begin{aligned}&l=\lim _{n}\lambda _n\Bigg \{\sum _{k \in {\mathcal {K}}} \int _{\Omega }\Bigg [ \sum _{w\in {\mathcal {W}}}\Bigg (\sum _{m\in {\mathcal {M}}}\frac{{\partial }c_{wh}^m(\omega ,y^*(\omega ))}{{\partial }y^k_{wh} }\Bigg )\times (y_{wh}^{k}(\omega ) -y^{*k}_{wh}(\omega )) \end{aligned}$$

41 $$\begin{aligned}&+ \frac{{\partial }\pi _h^k(\omega ,z_h^{*k}(\omega ))}{\partial z_h^k}\times (z_{h}^{k}(\omega ) -z^{*k}_{h}(\omega ))\Bigg ]dP(\omega )+\gamma _n\Bigg \}, \end{aligned}$$

42 $$\begin{aligned}&\theta _Y=\lim _n \lambda _n\Big (d_h^k(\omega )-\sum _{w\in {\mathcal {W}}} y^{k}_{wh}(\omega )-z^{k}_{h}(\omega )+u^1_n\Big ),\nonumber \\&\theta _Y=\lim _n \lambda _n\Big (\sum _{w\in {\mathcal {W}}}y^{k}_{wh}(\omega )+\sum _{w\in {\mathcal {W}}}x^{k}_{wh}- e_k(\omega )+u^2_n\Big ), \end{aligned}$$

43 $$\begin{aligned}&\theta _Y=\lim _n \lambda _n\Big (-y^{k}_{wh}(\omega )+u^3_n\Big ), \theta _Z=\lim _n \lambda _n\Big (-z^k_{h}(\omega )+u^4_n\Big ), \end{aligned}$$

44 $$\begin{aligned}&\theta _Z=\lim _n \lambda _n\Big (z^k_{h}(\omega )-\alpha d_h^k+u^5_n\Big ), \end{aligned}$$

with $$\gamma _n\ge 0$$, $$\lambda _n>0$$, $$u^i_n\ge 0$$, $$i=1,\ldots ,5$$, and

$$\begin{aligned}&\lim _{n}\Bigg \{\sum _{k \in {\mathcal {K}}} \int _{\Omega }\Bigg [ \sum _{w\in {\mathcal {W}}}\Bigg (\sum _{m\in {\mathcal {M}}}\frac{{\partial }c_{wh}^m(\omega , y^*(\omega ))}{{\partial }y^k_{wh} }\Bigg )\times (y_{wh}^{k}(\omega ) -y^{*k}_{wh} (\omega )) \\&\qquad \quad + \frac{{\partial }\pi _h^k(\omega ,z_h^{*k}(\omega ))}{\partial z_h^k}\times (z_{h}^{k}(\omega ) -z^{*k}_{h}(\omega ))\Bigg ]dP(\omega )+\gamma _n\Bigg \}=0,\\&\lim _n \Big (d_h^k(\omega )-\sum _{w\in {\mathcal {W}}} y^{k}_{wh}(\omega )-z^{k}_{h}(\omega )+u^1_n\Big )= \theta _Y,\\&\lim _n \Big (\sum _{w\in {\mathcal {W}}}y^{k}_{wh}(\omega )+\sum _{w\in {\mathcal {W}}}x^{k}_{wh}- e_k(\omega )+u^2_n\Big )= \theta _Y,\\&\lim _n \Big (-y^{k}_{wh}(\omega )+u^3_n\Big )= \theta _Y, \lim _n \Big (-z^k_{h}(\omega )+u^4_n\Big )=\theta _Z,\\&\lim _n \lambda _n\Big (z^k_{h}(\omega )-\alpha d_h^k+u^5_n\Big )=\theta _Z, \end{aligned}$$

then *l* must be nonnegative. We prove that every term in (40)–(41) tends to zero. We first considers only the terms in *y*:

$$\begin{aligned}&\lambda _n\Bigg \{\sum _{k \in {\mathcal {K}}} \int _{\Omega }\Bigg [ \sum _{w\in {\mathcal {W}}}\Bigg (\sum _{m\in {\mathcal {M}}}\frac{{\partial }c_{wh}^m(\omega , y^*(\omega ))}{{\partial }y^k_{wh} }\Bigg )\times \Big (y_{wh}^{k}(\omega ) -y^{*k}_{wh}(\omega )\Big )\Bigg \}\\&=\lambda _n\Bigg \{ \sum _{w \in {\mathcal {W}}} \sum _{k \in {\mathcal {K}}} \int _{\Omega ^{1k}_w} \nu _h^{1}(\omega )\Big (y_{wh}^{k}(\omega ) -y^{*k}_{wh}(\omega )\Big )dP(\omega )\\&+ \sum _{k \in {\mathcal {K}}} \int _{\Omega ^{2k}_w}\sum _{w\in {\mathcal {W}}} \Bigg (\sum _{m\in {\mathcal {M}}}\frac{{\partial }c_{wh}^m(\omega ,y^*(\omega ))}{{\partial }y^k_{wh} }\Bigg )(y_{wh}^{k}(\omega )-y_{wh}^{*k}(\omega ))dP(\omega )\Bigg \} \\&\ge \lambda _n\Bigg \{ \sum _{w \in {\mathcal {W}}} \sum _{k \in {\mathcal {K}}} \int _{\Omega ^{1k}_w} \nu _h^{1}(\omega )\Big (y_{wh}^{k}(\omega ) -y^{*k}_{wh}(\omega )\Big )dP(\omega )\\&+ \sum _{w \in {\mathcal {W}}} \sum _{k \in {\mathcal {K}}} \int _{\Omega ^{2k}_w} \nu _h^{1}(\omega )\Big (y_{wh}^{k}(\omega ) -y^{*k}_{wh}(\omega )\Big )dP(\omega ) \Bigg \}\\&=\lambda _n\Bigg \{ \sum _{w \in {\mathcal {W}}} \sum _{k \in {\mathcal {K}}} \int _{\Omega } \nu _h^{1}(\omega )\Big (y_{wh}^{k}(\omega ) -y^{*k}_{wh}(\omega )\Big )dP(\omega ) \Bigg \}\\&=\lambda _n\Bigg \{\sum _{k \in {\mathcal {K}}}\int _{\Omega } \nu _h^{1}(\omega )\Big ( \sum _{w \in {\mathcal {W}}} y_{wh}^{k}(\omega ) + \sum _{w \in {\mathcal {W}}} x_{wh}^k-e_k(\omega )+u_n^2\Big )dP(\omega )\\&+\sum _{k \in {\mathcal {K}}}\int _{\Omega } \nu _h^{1}(\omega )\Big (-\sum _{w \in {\mathcal {W}}}x_{wh}^k+e_k(\omega )-\sum _{w \in {\mathcal {W}}}y_{wh}^{*k}(\omega ) \Big )dP(\omega )\Bigg \}. \end{aligned}$$

Taking into account that $\sum _{w \in {\mathcal {W}}}y_{wh}^{*k}(\omega )=-\sum _{w \in {\mathcal {W}}}x_{wh}^k+e_k(\omega )$, all the terms tends to zero. Analogously, we can prove that the other terms tends to zero. $\square$

## Application of the Infinite-Dimensional Duality to the Second-Stage Problem

In this section, we prove that variational inequality (36) can be expressed in terms of Lagrange variables. As a consequence, the second-stage problem can be replaced by optimality conditions and the large-scale problem (9)–(17) can be reformulated.

### Theorem 5.1

$(y^*_h,z^*_h) \in T_h$ is a solution to (36) if and only if there exist $\lambda _{h}^{1k},\lambda _{h}^{2k},\mu _{w}^{1k},\mu _{w}^{2k},\mu _{w}^{3k}\in L^2(\Omega ,P, {\mathbb {R}}_+)$, such that

$$\begin{aligned}&\sum _{m\in {\mathcal {M}}}\frac{{\partial }c_{wh}^m(\omega ,y^*(\omega ))}{{\partial }y^k_{wh} }-\lambda _h^{*1k}(\omega )+\lambda _h^{*2k}(\omega )-\mu ^{*1k}(\omega )=0, {P-a.s. }\\&\frac{{\partial }\pi _h^k(\omega ,z_h^{*k}(\omega ))}{\partial z_h^k}-\mu ^{*2k}(\omega )+\mu ^{*3k}(\omega )=0, { P-a.s. }\\&{\lambda _{h}^{1k}}(\omega )\Big (d_h^k(\omega )-\sum _{w\in {\mathcal {W}}} y^{k}_{wh}(\omega )-z^k_{h}(\omega ) \Big )=0, { P-a.s. } \\&\lambda _{h}^{2k}(\omega ) \Big (\sum _{w\in {\mathcal {W}}}y^{k}_{wh}(\omega )+\sum _{w\in {\mathcal {W}}}x^{k}_{wh}- e_k(\omega )\Big )=0, {P-a.s. }\\&\mu _{w}^{1k}(\omega ) y_{wh}^k(\omega )=0,\, \mu _w^{2k}(\omega ) z_{h}^k(\omega )=0, { P-a.s. }\\&\mu _w^{3k}(\omega ) \Big (z_{h}^k(\omega )-\alpha d_h^k\Big )=0, { P-a.s. } \end{aligned}$$

### Proof

We assume that $(y^*_h,z^*_h) \in T_h$ is a solution to (36). For $h=1,\ldots ,H$ and for given $x\in S$, we set:

$$\begin{aligned} \Psi _h(x,y, z)= & {} \sum _{k \in {\mathcal {K}}} \int _{\Omega }\Bigg [ \sum _{w\in {\mathcal {W}}}\Bigg (\sum _{m\in {\mathcal {M}}}\frac{{\partial }c_{wh}^m(\omega ,y^*(\omega ))}{{\partial }y^k_{wh} }\Bigg )\times (y_{wh}^{k}(\omega ) -y^{*k}_{wh}(\omega )) \\&+ \frac{{\partial }\pi _h^k(\omega ,z_h^{*k}(\omega ))}{\partial z_h^k}\times (z_{h}^{k}(\omega ) -z^{*k}_{h}(\omega ))\Bigg ]dP(\omega ) \ge 0, \forall (y_h,z_h)\in T_h \end{aligned}$$

and observe that variational inequality (36) is equivalent to the minimization problem

45 $$\begin{aligned} \min _{y,z\in T}\Psi _h(x,y, z)=\Psi _h(x,y^*, z^*)=0. \end{aligned}$$

For $h=1,\ldots ,H$, we consider the Lagrange function associated with optimization problem (45):

46 $$\begin{aligned}&{\mathcal {L}}_h(x,y,z, \lambda ,\mu )= \Psi _h(x,y,z)\nonumber \\&+\int _0^T\sum _{k \in {\mathcal {K}}}{\lambda _{h}^{1k}}(\omega )\Big (d_h^k(\omega )-\sum _{w\in {\mathcal {W}}} y^{k}_{wh}(\omega )-z^k_{h}(\omega ) \Big )dP(\omega )\nonumber \\&+\int _0^T\sum _{k \in {\mathcal {K}}}\lambda _{h}^{2k}(\omega ) \Big (\sum _{w\in {\mathcal {W}}}y^{k}_{wh}(\omega )+\sum _{w\in {\mathcal {W}}}x^{k}_{wh}- e_k(\omega )\Big )dP(\omega ),\nonumber \\&-\int _0^T\sum _{k \in {\mathcal {K}}}\sum _{w \in {\mathcal {W}}} \mu _{w}^{1k}(\omega ) y_{wh}^k(\omega )dP(\omega )-\int _0^T\sum _{k \in {\mathcal {K}}} \mu _w^{2k}(\omega ) z_{h}^k(\omega )dP(\omega )\nonumber \\&+\int _0^T\sum _{k \in {\mathcal {K}}} \mu _w^{3k}(\omega ) \Big (z_{h}^k(\omega )-\alpha d_h^k\Big )dP(\omega ), \end{aligned}$$

$\forall y\in L^2(\Omega ,P,{\mathbb {R}}^{WH})$, $z\in L^2(\Omega ,P,{\mathbb {R}}^{HK})$, $\lambda _{h}^{1k},\lambda _{h}^{2k}, \mu _{w}^{1k},\mu _{w}^{2k},\mu _{w}^{3k}\in L^2(\Omega ,P,{\mathbb {R}}_+)$. Then, applying results in [5, 6], since we proved *Assumption* *S*, there exist $\lambda _{h}^{*1k}(\omega ),\lambda _{h}^{*2k}(\omega ),\mu _{w}^{*1k}(\omega ), \mu _{w}^{*2k}(\omega ),\mu _{w}^{*3k}(\omega )\ge 0$, *P*.a.s. such that $(y_h,z_h,\lambda _{h}^{*1k},\lambda _{h}^{*2k},\mu _{w}^{*1k},\mu _{w}^{*2k},\mu _{w}^{*3k})$ is a saddle point of the Lagrange functional

$$\begin{aligned}&{\mathcal {L}}_h(x,y^*,z^*, \lambda ,\mu ) \le {\mathcal {L}}_h(x,y^*,z^*, \lambda ^*,\mu ^*)\le {\mathcal {L}}_h(x,y,z, \lambda ^*,\mu ^*)\\&\forall (y_h,z_h)\in T_h, \forall \lambda _{h}^{1k}(\omega ),\lambda _{h}^{2k}(\omega ), \mu _{w}^{1k}(\omega ),\mu _{w}^{2k}(\omega ),\mu _{w}^{3k}(\omega )\ge 0, P.a.s.\\&{\lambda _{h}^{*1k}}(\omega )\Big (d_h^k(\omega )-\sum _{w\in {\mathcal {W}}} y^{*k}_{wh}(\omega )-z^{*k}_{h}(\omega ) \Big )=0,{ \,P-a.s. }\\&\lambda _{h}^{*2k}(\omega ) \Big (\sum _{w\in {\mathcal {W}}}y^{*k}_{wh}(\omega )+\sum _{w\in {\mathcal {W}}}x^{k}_{wh}- e_k(\omega )\Big )=0,{\, P-a.s. }\\&\mu _{w}^{*1k}(\omega ) y_{wh}^{*k}(\omega )=0,\, \mu _w^{*2k}(\omega ) z_{h}^{*k}(\omega )=0,{\, P-a.s. }\\&\mu _w^{*3k}(\omega ) \Big (z_{h}^{*k}(\omega )-\alpha d_h^k\Big )=0, {\, P-a.s. } \end{aligned}$$

Thus, we find

$$\begin{aligned}&0= {\mathcal {L}}_h(x,y^*,z^*, \lambda ^*,\mu ^*) \le {\mathcal {L}}_h(x,y,z, \lambda ^*,\mu ^*)\\&= \sum _{k \in {\mathcal {K}}} \int _{\Omega }\Bigg [ \sum _{w\in {\mathcal {W}}}\Bigg (\sum _{m\in {\mathcal {M}}}\frac{{\partial }c_{wh}^m (\omega ,y^*(\omega ))}{{\partial }y^k_{wh} }-\lambda _h^{*1k}(\omega ) +\lambda _h^{*2k}(\omega )-\mu ^{*1k}(\omega )\Bigg )\\&\times (y_{wh}^{k}(\omega ) -y^{*k}_{wh}(\omega ))\\&+ \Bigg (\frac{{\partial }\pi _h^k(\omega ,z_h^{*k}(\omega ))}{\partial z_h^k}-\mu ^{*2k}(\omega )+\mu ^{*3k}(\omega )\Bigg )\times (z_{h}^{k}(\omega ) -z^{*k}_{h}(\omega ))\Bigg ]dP(\omega ) \end{aligned}$$

Setting $y_{wh}^{k}(\omega )=y_{wh}^{*k}(\omega )\pm \epsilon _1(\omega )$, and then $z_{h}^{k}(\omega )=z_{h}^{*k}(\omega )\pm \epsilon _2(\omega )$, we find that

$$\begin{aligned}&\sum _{m\in {\mathcal {M}}}\frac{{\partial }c_{wh}^m(\omega ,y^*(\omega ))}{{\partial }y^k_{wh} }-\lambda _h^{*1k}(\omega )+\lambda _h^{*2k}(\omega )-\mu ^{*1k}(\omega )=0, \,{ P-a.s. }\\&\frac{{\partial }\pi _h^k(\omega ,z_h^{*k}(\omega ))}{\partial z_h^k}-\mu ^{*2k}(\omega )+\mu ^{*3k}(\omega )=0, \,{ P-a.s. } \end{aligned}$$

The converse is easily achieved. $\square$

Therefore, the two-stage problem can be reformulated as follows:

$$\begin{aligned}&\min \sum _{w\in {\mathcal {W}}}\Bigg (\sum _{k \in {\mathcal {K}}}\rho ^{k}_{w}x^{k}_{wh} +\sum _{m\in {\mathcal {M}}} t_{wh}^m({x})\Bigg )+ \int _{\Omega } \Phi _h(x,\xi (\omega ))dP(\omega )\\&\sum _{w\in {\mathcal {W}}}x^{k}_{wh}{\ge } d_h^k,\, \forall k\in {\mathcal {K}},\\&\sum _{w\in {\mathcal {W}}}x^{k}_{wh}\le e_k, \, \forall k\in {\mathcal {K}},\\&x^{k}_{wh}\ge 0,\, \forall w\in {\mathcal {W}},\, \forall k\in {\mathcal {K}}, \\&\sum _{m\in {\mathcal {M}}}\frac{{\partial }c_{wh}^m(\omega ,y^*(\omega ))}{{\partial }y^k_{wh} }-\lambda _h^{*1k}(\omega )+\lambda _h^{*2k}(\omega )\ge 0, { {P-a.s.}}\\&\frac{{\partial }\pi _h^k(\omega ,z_h^{*k}(\omega ))}{\partial z_h^k}+\mu ^{*3k}(\omega )\ge 0, { {P-a.s.}}\\&{\lambda _{h}^{1k}}(\omega )\Big (d_h^k(\omega )-\sum _{w\in {\mathcal {W}}} y^{k}_{wh}(\omega )-z^k_{h}(\omega ) \Big )=0, { {P-a.s.}}\\&\lambda _{h}^{2k}(\omega ) \Big (\sum _{w\in {\mathcal {W}}}y^{k}_{wh}(\omega )+\sum _{w\in {\mathcal {W}}}x^{k}_{wh}- e_k(\omega )\Big )=0, { {P-a.s.}}\\&\mu _w^{3k}(\omega )\Big (z_{h}^k(\omega )-\alpha d_h^k\Big )=0 , { {P-a.s.}} \end{aligned}$$

We now describe some relevant consequences that gives an insights into the market behavior with respect to the product shipment. Dual variables $\lambda _{h}^{1k}$, $\lambda _{h}^{2k}$, $\mu _{w}^{1k}$, $\mu _{w}^{2k}$, $\mu _{w}^{3k}$ regulate the medical item procurement. In particular, $\lambda _{h}^{1k}$ is a control variable on the first-stage demand; $\lambda _{h}^{2k}$ is a control variable on the item availability level; $\mu _{w}^{1k}$ is a control variable on the second-stage demand; $\mu _{w}^{2k}$ and $\mu _{w}^{3k}$ are control variables on the unfulfilled demand. We discuss some cases, considering active and non-active constraints. We have:

$$\begin{aligned} \sum _{m\in {\mathcal {M}}}\frac{{\partial }c_{wh}^m(\omega ,y^*(\omega ))}{{\partial }y^k_{wh}}-\lambda _h^{*1k}(\omega )+\lambda _h^{*2k}(\omega )-\mu ^{*1k}(\omega )=0, {{P-a.s.}} \end{aligned}$$

If $y^{*k}_{wh}(\omega )>0$, then $\mu _{w}^{1k}(\omega )=0$, *P-a.s.*, and

$$\begin{aligned} \sum _{m\in {\mathcal {M}}}\frac{{\partial }c_{wh}^m(\omega ,y^*(\omega ))}{{\partial }y^k_{wh}}= \lambda _h^{*1k}(\omega )-\lambda _h^{*2k}(\omega ) , \,{ P-a.s. } \end{aligned}$$

namely, the marginal cost is equal to the difference of the control variables on demand and market item availability. Moreover, if $\lambda _h^{*1k}(\omega )=0$, $\lambda _h^{*2k}(\omega )>0$, *P-a.s.*, we find $\sum _{m\in {\mathcal {M}}}\frac{{\partial }c_{wh}^m(\omega ,y^*(\omega ))}{{\partial }y^k_{wh} }=-\lambda _h^{*2k}(\omega ), { {P-a.s.}},$ and the marginal cost decreases. If $\lambda _h^{*2k}(\omega )=0$, $\lambda _h^{*1k}(\omega )>0$, *P-a.s.*, we find thet $\sum _{m\in {\mathcal {M}}}\frac{{\partial }c_{wh}^m(\omega ,y^*(\omega ))}{{\partial }y^k_{wh} }=\lambda _h^{*1k}(\omega ), { P-a.s. },$ and the marginal cost increases.

From

$$\begin{aligned} \frac{{\partial }\pi _h^k(\omega ,z_h^{*k}(\omega ))}{\partial z_h^k}=\mu ^{*2k}(\omega )-\mu ^{*3k}(\omega ), \text { P-a.s. }, \end{aligned}$$

we note that the marginal penalty is equal to the difference between the control variables on the unfulfilled demand. If $0<z_h^{*k}(\omega )<\alpha d_h^k, \,{ {P-a.s.}}$, then $\mu ^{*2k}(\omega )=\mu ^{*3k}(\omega )=0$, and $\frac{{\partial }\pi _h^k(\omega ,z_h^{*k}(\omega ))}{\partial z_h^k}=0, \,{ {P-a.s.}}$, namely, the marginal penalty is equal to zero. If $\mu ^{*2k}(\omega )>0$, then $z_h^{*k}(\omega )=0,\, {{P-a.s.}}$ This is the case of an effective emergency plan, in which hospital does not incur in any unmet demand. If $\mu ^{*3k}(\omega )>0$, then $\mu ^{*2k}(\omega )=0,\, { {P-a.s.}}$, and $\frac{{\partial }\pi _h^k(\omega ,z_h^{*k}(\omega ))}{\partial z_h^k}=-\mu ^{*3k}(\omega ), \, {{P-a.s.}}$, namely, the marginal penalty decreases.

## Numerical Example

In this section, we present two small numerical examples for illustrative purposes. We consider two warehouses ($w=2$), two hospitals ($h=2$), three different items ($k=3$), one transportation mode ($m=1$) and five scenarios. The economic data mainly come from [1]. For the calculation of transportation costs, we apply the Product & Distance-based calculation rule, which computes the transportation costs based on the coefficients for transportation costs (that include all the different terms, e.g., fuel price, tolls, etc.) for national shipments assumed to be . For transportation time from warehouses to hospitals, we consider hourly cost set, that includes the time spending for the loading process, the route to go and the unloading process. Penalty costs, concerning the unfulfilled demand, depends on the number of items that are not delivered in one day.

The numerical simulations are solved applying the Progressive Hedging Method (PHM) [31]. This is a well-known algorithm that has been recently extended to multistage SVI and multistage stochastic Lagrangian variational inequalities [33, 34]. In [2], the authors presented a new framework that shows how PHM can be utilized, while guaranteeing convergence, to globally optimal solutions of mixed-integer stochastic convex programs. We now briefly present the Progressive Hedging Algorithm for a two-stage stochastic optimization problem.

We consider the problem:

$$\begin{aligned} \min _{x\in X} f(x)+ {\mathbb {E}}_{\xi }(\Phi _h(x,\xi (\omega ))), \end{aligned}$$

where

$$\begin{aligned} f(x)=\sum _{w\in {\mathcal {W}}}\Bigg (\sum _{k \in {\mathcal {K}}}\rho ^{k}_{w}x^{k}_{wh} +\sum _{m\in {\mathcal {M}}} t_{wh}^m(x)\Bigg ) \end{aligned}$$

is convex in *x* and $\Phi _h(x,\xi (\omega ))$ is the recourse function, defined as the second-stage optimal value function

$$\begin{aligned} \Phi _h(x,\xi (\omega ))=\min _{y(\xi ) \in Y(x,\xi )} g(x,y(\xi ),\xi (\omega )), \end{aligned}$$

where

$$\begin{aligned} g(x,y(\xi ),\xi (\omega ))=\sum _{w\in {\mathcal {W}}}\sum _{m\in {\mathcal {M}}}c_{wh}^m(\omega ,y(\omega ))+\sum _{k\in {\mathcal {K}}}\pi _{h}^k(\omega , z^k_{h}(\omega )), \end{aligned}$$

$c_{wh}^m(\omega ,\cdot )$ and $\pi _{h}^k(\omega ,\cdot )$ are convex function for all *w*, *h*, *k*, *m*. 
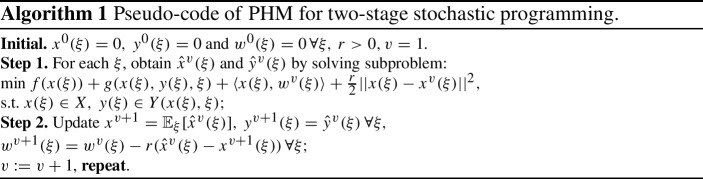


We emphasize that the convergence of PHM to global optimal solution is ensured for convex stochastic programs if the involved function in the corresponding variational inequality is strongly monotone, [33, 34].

All the codes were written in MATLAB and run in MATLAB R2020a (derived data supporting the findings of this study are available from the corresponding author upon request.). Following a discrete approximation scheme as in [23], we choose $|R|=5$ realizations of random variable $\xi$ with probability 1/*R*. We note that hospital medical items are generally purchased as multiple packs into boxes. In our examples, we consider two different cases. In the first example, we consider some indispensable items; hence, we use a high penalty of unfulfilled demand, and we consider all cost referred to a single pack. In the second example, we consider a box as a unit of measurement, which contains thousand packs and a low penalty of unfulfilled demand.

Numerical Example 1: In this numerical example, the items are collected in multiple packages, and the coefficients of the cost functions are related to a single package. We have considered high penalty functions, since not satisfying the demand for a particular item would cause severe discomfort. It can be noted that, given the danger of the penalty, at equilibrium we get zero penalties as it is likely that hospitals pay more attention to some indispensable items. The matrix of the cost functions is given by

47 $$\begin{aligned} \begin{pmatrix} 2,6 &{} \quad 2,9 &{}\quad 3,5 &{}\quad 7,2 &{}\quad 0,00019 &{}\quad 0,00029 &{}\quad 0,00038 &{} \quad 0,00023 &{}\quad 1800 &{}\quad 1600\\ 2,7 &{} \quad 2,5 &{}\quad 1,7 &{}\quad 2,2 &{}\quad 0,00025 &{}\quad 0,00031 &{}\quad 0,00024 &{}\quad 0,00018 &{}\quad 1500 &{}\quad 1400 \\ 1,6 &{} \quad 1,9 &{} \quad 1,02 &{}\quad 2,03 &{}\quad 0,00021 &{}\quad 0,00036 &{}\quad 0,00032 &{} \quad 0,00028 &{}\quad 1300 &{}\quad 1500 \end{pmatrix}. \nonumber \\ \end{aligned}$$

First of all, we focus our attention on the flows $x_{wh}^k$ of the first stage. We find

48 $$\begin{aligned} x^1_{11}= & {} 3,00; \quad x^1_{12}=3,40; \quad x^1_{21}=0,00; \quad x^1_{22}=0,00;\nonumber \\ x^2_{11}= & {} 0,00; \quad x^2_{12}=0,00; \quad x^2_{21}=0,00; \quad x^2_{22}=0,00;\nonumber \\ x^3_{11}= & {} 0,00; \quad x^3_{12}=1,50; \quad x^3_{21}=1,00; \quad x^3_{22}=0,00. \end{aligned}$$

From the numerical result of the first example [see (48),Table 2], we notice that in a condition without emergency, each hospital prefers to choose his trusted warehouse. In particular,

- All hospitals decide to buy the medical item one $(k=1)$ from warehouse one $(w=1)$;
- All hospitals decide not to buy the medical item two $(k=2)$ from warehouse one $(w=1)$ or two $(w=2)$;
- For medical item three $(k=3)$, hospital two $(h=2)$ decides to rely on warehouse one $(w=1)$ and hospital one $(h=1)$ on warehouse two $(w=2)$.

In the second stage, namely, in an emergency situation, the usual choice is no longer the optimal one, but demand must always be satisfied by minimizing costs. Furthermore, the penalties are fortunately null for each hospital and for each item. The results are shown in Table 2.

**Table 2:** Numerical results solved by PHM about indispensable items

Items	Flows	Scenario1	Scenario2	Scenario3	Scenario4	Scenario5
$k=1$	$y_{11}(\omega )$	1,21	1,56	1,66	1,82	2,04
	$y_{12}(\omega )$	1,12	1,12	1,31	1,41	1,57
	$y_{21}(\omega )$	0,79	0,64	0,85	0,99	1,03
	$y_{22}(\omega )$	1,29	1,49	1,58	1,68	1,91
$k=2$	$y_{11}(\omega )$	0,51	0,62	0,77	0,91	1,01
	$y_{12}(\omega )$	0,56	0,57	0,73	0,81	0,85
	$y_{21}(\omega )$	0,51	0,65	0,81	0,94	1,06
	$y_{22}(\omega )$	0,65	1,00	1,16	1,28	1,42
$k=3$	$y_{11}(\omega )$	0,34	1,25	0,48	0,57	0,79
	$y_{12}(\omega )$	0,44	0,46	0,54	0,58	0,64
	$y_{21}(\omega )$	0,31	0,27	0,37	0,40	0,41
	$y_{22}(\omega )$	0,48	0,55	0,66	0,72	0,87
$k=1$	$z_{1}(\omega )$	0,00	0,00	0,00	0,00	0,00
	$z_{2}(\omega )$	0,00	0,00	0,00	0,00	0,00
$k=2$	$z_{1}(\omega )$	0,00	0,00	0,00	0,00	0,00
	$z_{2}(\omega )$	0,00	0,00	0,00	0,00	0,00
$k=3$	$z_{1}(\omega )$	0,00	0,00	0,00	0,00	0,00
	$z_{2}(\omega )$	0,00	0,00	0,00	0,00	0,00

*Numerical Example 2:* In this numerical example, the items are treated as boxes and the coefficients of the cost functions are related to boxes which contain thousand packages. We have considered low penalty functions, since not satisfying the demand for a particular item would not cause severe discomfort. In this case, the amount of unfulfilled demand at hospital *h* of medical supply item *k* under scenario $\omega$, for all *h*, *k* are not null. This is a consequence of the fact that for these items it is not necessary to satisfy fully the daily demand. Another difference with the first numerical example is that all hospitals use all warehouses, without choosing the trusted warehouses.

The coefficient matrix of the cost functions is represented by (49).

49 $$\begin{aligned} \begin{pmatrix} 2,41 &{} \quad 2,44 &{}\quad 2,47 &{}\quad 2,42 &{}\quad 1,90 &{}\quad 2,85 &{}\quad 3,80 &{} \quad 2,28 &{}\quad 0,05 &{}\quad 0,04\\ 2,81 &{} \quad 2,84 &{}\quad 2,87 &{}\quad 2,82 &{}\quad 2,54 &{}\quad 3,10 &{}\quad 2,42 &{}\quad 1,83 &{} \quad 0,07 &{}\quad 0,03\\ 1,76 &{} \quad 1,79 &{} \quad 1,82 &{}\quad 1,77 &{}\quad 2,10 &{}\quad 3,63 &{}\quad 3,21 &{} \quad 2,82 &{}\quad 0,02 &{}\quad 0,08\\ \end{pmatrix}\times 10^{-3}.\qquad \end{aligned}$$

The flows $x_{wh}^k$ of the first stage (50) are given by

50 $$\begin{aligned} x^1_{11}&=1,65; \quad x^1_{12}=1,71; \quad x^1_{21}=1,44; \quad x^1_{22}=1,78; \nonumber \\ x^2_{11}&=1,14; \quad x^2_{12}=1,11; \quad x^2_{21}=0,94; \quad x^2_{22}=1,17; \nonumber \\ x^3_{11}&=0,64; \quad x^3_{12}=0,79; \quad x^3_{21}=0,52; \quad x^3_{22}=0,85. \end{aligned}$$

In Table 3, we group all variables for the second stage, under scenario $\omega$.

**Table 3:** Numerical result solved by PHM with unfulfilled demand

Items	Flows	Scenario1	Scenario2	Scenario3	Scenario4	Scenario5
$k=1$	$y_{11}(\omega )$	1,25	1,47	1,77	2,07	2,22
	$y_{12}(\omega )$	0,44	0,51	0,46	0,51	0,42
	$y_{21}(\omega )$	0,21	0,15	0,15	0,15	0,21
	$y_{22}(\omega )$	1,25	1,38	1,63	1,79	2,32
$k=2$	$y_{11}(\omega )$	0,34	0,39	0,51	0,63	0,70
	$y_{12}(\omega )$	0,10	0,20	0,21	0,21	0,24
	$y_{21}(\omega )$	0,41	0,46	0,64	0,80	0,94
	$y_{22}(\omega )$	0,75	0,94	1,22	1,41	1,57
$k=3$	$y_{11}(\omega )$	0,37	0,44	0,52	0,62	0,72
	$y_{12}(\omega )$	0,19	0,23	0,25	0,28	0,23
	$y_{21}(\omega )$	0,14	0,17	0,19	0,19	0,22
	$y_{22}(\omega )$	0,50	0,52	0,69	0,75	0,96
$k=1$	$z_{1}(\omega )$	0,65	0,68	0,67	0,67	0,68
	$z_{2}(\omega )$	0,79	0,78	0,79	0,78	0,77
$k=2$	$z_{1}(\omega )$	0,40	0,44	0,44	0,44	0,44
	$z_{2}(\omega )$	0,47	0,47	0,48	0,48	0,48
$k=3$	$z_{1}(\omega )$	0,21	0,20	0,20	0,20	0,19
	$z_{2}(\omega )$	0,32	0,32	0,33	0,33	0,34

## Conclusions

In this paper, we constructed a stochastic Nash equilibrium model for a medical supply network that consists of warehouses and hospitals with multiple medical items and multiple transportation modes. Each hospital solves a two-stage stochastic optimization problem, where, in the first stage, seeks to minimize the purchasing cost of medical items and the transportation time. Then, we introduced a recourse decision process to optimize the expected overall costs and the penalty for the prior plan, in response to each possible disaster scenario of the second stage. The hospitals simultaneously solve their own stochastic optimization problems and reach a stable state given by the stochastic Nash equilibrium concept. Specific features of the model include: the uncertainty of the scenarios, the supply availability of medical items, the penalty for unmet demand and the fluctuating costs. The model is formulated as a variational inequality. In the case of general probability distribution, we characterized the Nash equilibrium of the problem as a solution to an infinite-dimensional variational inequality in the Hilbert space $L^2$. The associated Lagrange function was studied, and a strong duality result was provided. Finally, we presented some numerical illustrations solved applying the progressive hedging algorithm.

The results reveal that hospitals are able to re-arrange timely their requests in order to satisfy the need for medical items. In emergencies, uncertainty plays a fundamental role in the success of disaster management; hence, health institutions must be ready to adjust the request of medical items. Our contributions to the literature lie in advancing the state-of-the-art of stochastic programming for disaster management as well as applications of variational inequalities and strong duality. We also emphasize that to date there has been limited work on stochastic programming problems under general probability distribution.

This model could be extended in future research. For example, we could incorporate additional details to the model and solve examples using data from real situations. The extension to a multi-stage problem where we consider different stages of information is another future research opportunity.
